# Depression and Quality of Life among Patients Living with HIV/AIDS in the Era of Universal Treatment Access in Vietnam

**DOI:** 10.3390/ijerph15122888

**Published:** 2018-12-17

**Authors:** Bach Xuan Tran, Anh Kim Dang, Nu Thi Truong, Giang Hai Ha, Huong Lan Thi Nguyen, Ha Ngoc Do, Tuan Quoc Nguyen, Carl A. Latkin, Cyrus S. H. Ho, Roger C. M. Ho

**Affiliations:** 1 Institute for Preventive Medicine and Public Health, Hanoi Medical University, Hanoi 100000, Vietnam; 2 Bloomberg School of Public Health, Johns Hopkins University, Baltimore, MD 21205, USA; carl.latkin@jhu.edu; 3 Institute for Global Health Innovations, Duy Tan University, Da Nang 550000, Vietnam; kimanh.ighi@gmail.com (A.K.D.); giang.ighi@gmail.com (G.H.H.); huong.ighi@gmail.com (H.L.T.N.); 4 Center of Excellence in Behavioral Medicine, Nguyen Tat Thanh University, Ho Chi Minh City 700000, Vietnam; nu.coentt@gmail.com (N.T.T.); hocmroger@yahoo.com.sg (R.C.M.H.); 5 Youth Research Institute, Viet Nam (YRI)-Ho Chi Minh Communist Youth Union, Hanoi 100000, Vietnam; ngochayri@gmail.com; 6 Hanoi Department of Health, Hanoi 100000, Vietnam; quoctuan3172@yahoo.com; 7 Department of Psychological Medicine, National University Hospital, Singapore 119074, Singapore; cyrushosh@gmail.com; 8 Department of Psychological Medicine, Yong Loo Lin School of Medicine, National University of Singapore, Singapore 119228, Singapore

**Keywords:** depression, quality of life, CD4 cells, early antiretroviral treatment initiation, HIV/AIDS, Vietnam

## Abstract

Although antiretroviral treatment (ART) access has been universal in recent years, few studies have examined if this policy contributes to the mental health of the patients. This study assessed depression and its relations with health-related quality of life (HRQOL), which is defined as the status of general well-being, physical, emotional, and psychological, among HIV patients. A cross-sectional study was conducted in 482 patients at five outpatient clinics. Patient Health Questionnaire-9 (PHQ-9) and EuroQol-5 dimensions-5 levels (EQ-5D-5L) were used to assess the severity of depression and HRQOL. About one-fifth of patients reported symptoms of depression. According to the result of a multivariate logistic regression model, patients who had a lower number of CD4 cells at the start of ART, who received ART in the clinic without HIV counseling and testing (HCT) services, who had a physical health problem, and who experienced discrimination were more likely to have depression. Depression was associated with significantly decreased HRQOL. Depression is prevalent and significantly negatively associated with HRQOL of HIV/AIDS patients. We recommend screening for depression and intervening in the lives of depressed individuals with respect to those who start ART late, and we also recommend community-based behavioral change campaigns to reduce HIV discrimination.

## 1. Introduction

Depression has become one of the most common psychiatric disorders and negatively affects adherence to and outcomes of antiretroviral treatment (ART) [1]. Throughout the course of HIV infection, patients may experience depressive symptoms due to deteriorated health status, difficulty functioning, ART side effects, apathy, stigma, and discrimination [2,3,4]. Conversely, people who suffer from depression may have increased risk of substance abuse, unsafe injection [5], and engagement in high-risk sexual behaviors [6] that fuel the spread of HIV epidemics.

The proportion of depression among HIV/AIDS patients receiving ART in resource-constrained settings has been high. For example, it was 45.8% in Ethiopia in 2013 [7], 47% in Uganda in 2006 [8], and 25.4% in South Africa in 2012 [2]. Several positive drivers related to depression were identified, such as poorer socioeconomic background, gender inequalities [7], and self-isolation as a result of the community’s stigma and discrimination [9]. In addition to psychotherapy and pharmacotherapy, previous studies have also highlighted social factors that reduce depression among HIV/AIDS patients, for instance, greater support from family members and peer educators as well as socioeconomic empowerment, especially for women [7,10,11].

Although depression has been well-known as a predictor of poor ART adherence and outcomes, the extent to which this health issue has been prioritized and how interventions incorporated into care and treatment services are very limited in developing countries. Over the course of ART, a large proportion of depressed patients have reported suboptimal adherence, including missing doses and appointments with doctors [12,13]. Some studies have found an association between depression and increased risks of HIV transmission [14] and disease progression [15], as well as suicidal ideation [16]. In HIV epidemics largely driven by drug injection, depression was not only affecting the outcomes of care and treatment services but also was a major reinforcing factor contributing to engagement in many risk behaviors and to multiple health and social issues among drug users [17,18].

The HIV epidemic in Vietnam is still in a concentrated stage in which most-at-risk populations include commercial sex workers, drug users, and men who have sex with men (MSM). In 2016, the estimated coverage of ART services was 48% [19] and the country adapted the WHO Guideline on Universal Treatment Access for patients with HIV/AIDS, which showed that all HIV-infected patients can start having ART as soon as possible with any CD4 cell count and at any clinical stage [20]. This policy has the potential to improve physical health outcomes and bring hope to such patients as well as reduce the risk of HIV transmission at the population level given the suppressed viral load [20]. Although ART coverage has been rapidly expanded, psychological and social disorders have continued to affect the lives of patients, and effective interventions are still very limited. It has been documented that early ART initiation may also lead newly diagnosed HIV patient to a fear of HIV disclosure, a higher probability of stigma and discrimination, and a higher risk of suffering from potential side effects of the ART [21]. Several assessments of depression among patients with HIV/AIDS showed heterogeneity in results. A study conducted in Ho Chi Minh City using the Center for Epidemiologic Studies—Depression scale showed that 36.5% of participants had clinical depression [22]. The result of another study examining the prevalence of depression among men having HIV infection depicted that the depression rate was 18.7% over a 1-month period, measured by The Phan Vietnamese Psychiatric Scale [23]. A study of HIV patients receiving ART and attending two HIV outpatient clinics in Hanoi estimated that 26.2% of respondents experienced depression, based on the Center for Epidemiologic Studies—Depression scale [24]. Consequently, how early initiation of ART influences psychological well-being, such as HRQOL, which is defined as the status of general well-being, be it physical, emotional, or psychological [25], is still not well-established, especially in the resource-limited settings as Vietnam. The purpose of our study was to examine the prevalence of depression and its relation to the HRQOL of HIV/AIDS patients in various clinics in different geographical locations of Vietnam.

## 2. Materials and Methods

### 2.1. Study Setting and Sampling Method

A cross-sectional study was conducted from July to September 2017 in Hanoi, Lao Cai, and Thanh Hoa, three Vietnamese epicenters providing HIV/AIDS treatment services in the north of Vietnam. Participants were interviewed in five outpatient clinics: one provincial center (Thanh Hoa Provincial HIV/AIDS Control Center), three district general hospitals (Quang Xuong in Thanh Hoa, Bao Thang in Lao Cai and Ba Vi in Hanoi), and one district health center (Ung Hoa in Hanoi).

Convenience sampling was used to recruit participants. The eligibility criteria for choosing participants included the following: (1) being 18 years old or above; (2) agreeing to participate in the study; (3) having ART at aforementioned clinics; and (4) being present at the time of interviewing. We invited participants to a counseling room and clearly explained the purpose of the study. Written informed consent was given, and the confidentiality of their participation was ensured if participants agreed to involve to the study. There were 482 participants that agreed to enroll in this research.

### 2.2. Measurements and Instruments

We collected data through face-to-face interviews using interview-administered questionnaires.

#### 2.2.1. Socioeconomic Characteristics

The socioeconomic characteristics variables were as follows: gender; age measured as a continuous variable; marital status including single, living with spouse or partner, and divorce; academic level classified as under high school, high school, and above high school; occupation including unemployed, self-employed, and employed; and study site mentioned as above.

#### 2.2.2. Patient Health Questionnaire-9 (PHQ-9)

In order to determine the levels of depression severity among ART patients, we used a validated depression screening tool named PHQ-9, which is based on the Diagnostic and Statistical Manual of Mental Disorders (DSM-IV) criteria, including nine items. Each question would be pointed from 0 to 3, and the total score would be ranged from 0 to 27. The severity of depression was characterized as none (0–4 points), mild (5–9 points), moderate (10–14 points), moderately severe (15–19 points), and severe (≥20 points). Major depressive syndrome was defined if the first question scored 3 and five questions from first to fifth scored 2 or 3; it was counted as major depressive syndrome whenever the last question about suicidal ideation presented [26].

#### 2.2.3. Health Status

Health-related quality of life among participants was assessed by the EQ-5D-5L questionnaire, including five items with five levels of severity: mobility, self-care, usual activities, pain/discomforts, and anxiety/depression. A Vietnamese translation of the EQ-5D-5L has been developed and validated elsewhere [27]. EQ-5D-5L establishes 3125 health states, which are measured by a single index that ranges from −1 to 1. In order to self-assess the quality of life of participants, we utilized a visual analog scale (EQ-VAS) in which the total score ranges from 0 points (the worst health that you can imagine) to 100 points (the best health that you can imagine).

#### 2.2.4. ART-Related Characteristics

Participants self-reported their HIV stages (symptomatic, asymptomatic, and AIDS), ARV duration, and satisfaction toward treatment results. The study also examined whether each study site provided HIV counseling and testing (HCT) services or not. The CD4 cell count at the start of ART and the last time that testing was recorded from the participants’ medical records. According to the Guideline for HIV Treatment and Care in 2015 of the Vietnamese Ministry of Health, the threshold of CD4 cell count for patients to receive ART was <500 cell/μL [28]. In 2017, the guideline changed such that HIV patients should take ART as soon as possible at any clinical stage and with any CD4 cell count [20]. Therefore, in this study, we considered early ART initiation to be when participants had a CD4 cell count of ≥500 cell/μL at the time of ART.

#### 2.2.5. Health Risk Behaviors

High alcohol consumption and abuse were measured by the Alcohol Use Disorders Identification Test-Consumption (AUDIT-C). A total score for each answer ranged from 0 to 12. The higher the AUDIT-C score, the more likely it was that participants had alcohol abuse or dependence. The cutoff for identifying hazardous drinking was 4 in men and 3 in women. If participants answered positive responses regarding “How often do you have six or more drinks on one occasion?” they were classified as binge drinkers. We defined participants as smokers if they smoked at least 100 cigarettes during their lifetime.

We also asked participants “Have you ever used a drug in your lifetime?”

#### 2.2.6. Stigma/Discrimination

Stigma was measured based on four dimensions: (1) blame/judgment, (2) shame, (3) fear of transmission of HIV to others, and (4) health status disclosure [29]. Participants were asked whether they suffered from any type of stigma mentioned above or discrimination in multiple settings (family, workplace, health care facilities, and community) within the last month [29]. In addition, the participants were asked about deriving support from their peers (for instance, ART adherence, the information regarding treatment and care, health services, and work) during ART (Yes/No) and about sharing their health status with their peers (Yes/No).

### 2.3. Statistical Analysis

Data were analyzed by STATA version 12 (Stata Corp. LP, College Station, TX, USA). We used a chi square and a Mann–Whitney test to analyze the differences regarding health status, health risk behavior, and ART-related characteristics between depression and non-depression groups. We examined the factor that was associated with depression by the multivariate logistic regression model. Because measured domains were continuous variables, the multivariate Tobit regression was applied to determine factors that were associated with the PHQ-9 score and one’s quality of life. The forward stepwise selection strategy was also applied to exclude the non-significant variables, and the threshold to select variables for reduced models was 0.2. A p-value of the log likelihood ratio test <0.01 was the cutoff for statistical significance. In this study, we also considered 0.05 as the threshold for statistical significance.

### 2.4. Ethics Approval

The study protocol was reviewed and approved by the IRB of the Hanoi Medical University. Participants’ information was only used for research and kept confidentially (ethic approval code: 23THS17/HDDDDHYHN).

## 3. Results

A total of 482 patients enrolled in the study. Table 1 shows that most of the participants were male (61.3%) and living with a spouse/partner (66.6%). The majority of respondents had not completed high school (66.5%). The percentage of patients who were unemployed in the depression group was higher than that of the non-depression group (17.7% and 7.1% respectively). Among the study sites, the proportion of depressive participants was higher than the counterpart at the Thanh Hoa HIV/AIDS Control Center, Quang Xuong District General Hospital, and Ba Vi District General Hospital, and the differences were statistically significant. The mean age of participants was 38.4 (SD = 8.3).

The majority of respondents were asymptomatic (82.7%) (Table 2). The proportion of respondents satisfied with treatment results in the depression group was significantly lower than that in the non-depression group (85.1% vs. 94.8%). Approximately half of the patients were smokers (53.6%) and drug users (44%), and the percentage of participants who were drug users in the depression group was statistically higher than that in the non-depression group (55.2% and 41.2%, respectively). About 60% of the participants received ART in the clinic integrating HCT services (58.5%). Approximately 30% of participants received early ART initiation with a CD4 cell count ≥500. The average duration of ART was 3.8 years (SD = 2.5). The mean number of CD4 cell measurements at the start of ART and the last test of treatment duration was 389 (SD = 252) and 455 (SD = 244).

Table 3 illustrates that half of the respondents were not depressed (49.1%) and approximately one-third of patients had mild depressive symptoms (30.8%). Among respondents suffering from major depressive syndrome (20.2%), 12.8% had moderate depressive symptoms, 4.6% had moderate severe depressive symptoms, and 2.7% had severe depressive symptoms. 

Health-related quality of life among respondents is depicted in Table 4. The percentage of patients reporting any problems in mobility, self-care, usual activities, pain/discomfort, and anxiety/depression in the depression group was statistically significantly higher than those who had health problems in the non-depression group. The mean of total EQ-5D utility scores was 0.8 (SD = 0.2), and the EQ-5D index in the depression group was less statistically significantly than that in the non-depression group (0.6 and 0.8, respectively). In addition, the mean EQ-VAS score was 73.6 (SD = 15.3) with a statistically higher score in the non-depression group compared to the depression group (76.4 and 62.1 respectively).

The prevalence of stigma and discrimination among ART patients is shown in Table 5. The study reveals that the proportions of patients who felt shame about their health status were higher in the depression group compared to the non-depression group. Most of the respondents also experienced discrimination in the community (69.8%), and nearly fifty percent of patients were under the fear of HIV transmission to others (46.1%). Two-thirds of participants received support from peers and shared their health information with them.

In Table 6, regarding clinical characteristics, participants who had a CD4 cell count from 350 to 500 were more likely to have depression compared to those who had a CD4 cell count ≥500. In addition, a higher likelihood of depression was associated with participants having ART in a clinic that did not provide HCT services. Being in a symptomatic stage and having a higher AUDIT score were positively associated with depression, while participants who were satisfied with treatment results were less likely to suffer from depression. In terms of health status, having problems with mobility and performing daily activities and suffering from pain and discomfort were related to a higher risk of depression among ART patients. Furthermore, regarding stigma and discrimination, depression was also positively associated with those who experienced discrimination in the workplace and in the community and negatively associated with disclosed health status with family.

Table 7 indicates the various factors associated with HRQOL among participants. Those having depression were more likely to have a lower quality of life according to both the EQ-5D-5L index and EQ-VAS. Moreover, being unemployed, experiencing discrimination, and having complications and concurrent disease increased the likelihood of having a lower quality of life.

## 4. Discussion

Findings of the study provide valuable information about depression among HIV/AIDS patients in the era of early universal ART access in Vietnam. We found that about one-fifth of participants had depression. Participants who had a lower CD4 cell count at the start of ART or who received ART at the clinic without HCT services were more likely to suffer from depression. In addition, having a physical health problem and suffering from discrimination in the workplace and community were also positively associated with depression. By contrast, satisfaction with treatment results and disclosing health status with family were factors that decreased the depression score. Depression was also associated with a higher risk of a low quality of life. 

The percentage of participants having depression in our study was lower than that of other studies in Vietnam [22,24] and China [30]. The difference can be explained by the fact that our study was conducted among facilities from different levels of health systems in urban, rural, and mountainous areas, while other studies were carried out in urban areas only. In addition, in order to assess depression among participants, we used the PHQ-9 questionnaire, while other studies used different instruments such as Center for Epidemiologic Studies—Depression scale and The Phan Vietnamese Psychiatric Scale.

Participants who had a lower CD4 cell count at the first time of receiving ART were more likely to have depression compared to those with a CD4 cell count ≥500. This can be explained by the fact that early initiate ART can improve health status, delay the early onset of co-morbidity, decrease the adjusted mortality rates compared to those who defer ART until reaching CD4 <500 cells/μL [31], and therefore improve mental health and quality of life of HIV/AIDS patients [32]. On the other hand, individuals with depressive symptoms may diminish the effort to receive HIV care and initiate ART [33,34] due to cognitive impairment and self-isolation [35]. In addition, receiving ART in the clinic without HCT services also increases the risk of suffering from depression. Typically, HCT consists of pretest and posttest counseling sessions that help individuals to understand their personal health status and make informed choices based on their knowledge [36]. Furthermore, users are also offered information related to psychosocial support [37], and adequate ongoing counseling can prevent serious or prolonged psychological problems as well as avoid stigma and discrimination [38].

Compared to previous studies, we found similar results that indicate that the depression scores were higher among participants who were in the symptomatic stage compared to those who were in the asymptomatic stage [39,40]. This can be explained by the fact that patients with a more severe and progressive illness would be more likely to be depressed. During the symptomatic stage, patients have to cope with residual opportunistic infections, the deterioration of the immune system, and the side effects of ART medication [27].

Most depressed participants in the study had physical health problems. In particular, those suffering from pain and discomfort had a significantly higher risk of depression compared to who did not, and this finding is consistent with a previous study [41]. Patients who undergo pain may have depression for a longer period and with greater severity compared to those who do not [42]. HIV patients, especially those who are in a symptomatic or AIDS stage, may consider pain as a symptom of depression or as an aversion that triggers an extremely negative reaction such as depression [41,43]. Conversely, the depressive disorder can forecast the development of pain due to the somatization of emotional problems [44]. In addition, the results of multivariate regression analysis also indicate that patients who have a higher AUDIT-score have a greater likelihood of depression. The relationship between alcohol and depression is bi-directional, meaning that people who are depressed may abuse substances to lift their mood and escape feelings of despair [45]. Conversely, those who abuse substances are also more likely to suffer from depression due to the withdrawal process or the consequence of repeated use [46].

In Vietnamese settings, discrimination and stigma are considered as significant barriers that affect ART outcomes [11]. In our study, participants who suffered from discrimination in the workplace were more likely to be depressed compared to those who did not. This finding is consistent with previous studies, which indicate that self-stigma and discrimination may contribute to the development of depressive symptoms [47,48]. The majority of participants were self-employed and blue-collar-workers who were more likely to perceive a discriminatory environment compared to white-collar workers [49]. According to the “People Living with HIV Stigma Index” report in 2012, HIV patients lost their job or source of income, were refused opportunities to work, and experienced discriminatory attitudes from their colleagues who became aware of their HIV status as a result of HIV infection [50]. Therefore, being blocked access to daily activities and undergoing prejudice may trigger negative attitudes as well as depression among people living with HIV/AIDS. Furthermore, disclosing one’s health status to family members was negatively associated with depression. This finding is consistent with previous studies that showed that the majority of participants reported disclosure as a positive event for both them and family [51]. Several psychological disorders and behavioral issues may be increased by the lack of health status disclosure [52,53].

Our study results also suggest a negative association between depression and quality of life. This finding is similar to previous studies in which it was observed that the more severe the depressive symptoms, the poorer the quality of life scores were [54] and that symptoms of depression may negatively impact quality of life in various evaluated dimensions [55]. A study of Multicenter AIDS Cohort Studies in 2006 also revealed that lower mental health measured by the SF-36 questionnaire was significantly related to an increased depression score [56]. Moreover, results of a six-month follow-up study illustrate that hopelessness, which is considered a major symptom of depression, predicted a poor quality of life [57].

Several implications can be drawn from this study. Since depression was less prevalent among those initiating ART early, depression screenings and interventions should be targeted at participants who started ART late. A comprehensive health care program that integrates HCT delivery models with ART should be developed, and information regarding psychological support during treatment procession for HIV/AIDS patients should be provided. ART patients should be encouraged to share their health status and derive support from family members, reducing the risk of depression. From a community perspective, as a high level of discrimination was positively associated with depression, community-based legal support services and educational campaigns based on behavioral changes should be prioritized to address the myths and beliefs regarding stigma and discrimination among HIV/AIDS patients.

The strengths of this study include the recruitment of patients from many levels of health systems in rural, urban, and mountainous areas. Several international instruments were also employed, such as PHQ-9 and EQ-5D-5L, in order to help to increase the comparability between these study findings and worldwide studies. However, we should acknowledge some limitations in this study. Firstly, the convenience sampling technique was conducted to recruit participants, which may limit the generalizability of our results to other study populations. Secondly, because of the self-reported information, the data is susceptible to the recall bias of interviewers and to social desirability. In order to minimize biases, we removed the interviewers who were affiliated with selected health centers, and we also explained the instructions as well as the benefits and drawbacks to participants once they became involved in the study. Finally, because our study design was cross-sectional, causal inferences could not be made.

## 5. Conclusions

In conclusion, the study depicts that one-fifth of ART patients suffer from depression. Our study also emphasized the association between the higher likelihood of depression and both starting ART late and receiving ART in a clinic without HCT services. In addition, having physical health problem and suffering from discrimination in the workplace and community were also positively associated with depression. The depression was associated with a higher risk of a low quality of life. Depression screening and intervening should be conducted among those starting ART late. A comprehensive health care program integrating HCT delivery models with ART should be developed, and mass community-based behavioral change campaigns about HIV/AIDS awareness should be prioritized in order to reduce the risk of depression.

## Figures and Tables

**Table 1:** Demographic characteristics of patients receiving antiretroviral treatment (ART) (*n* = 482).

Characteristics	Depression						*p*-Value
	Yes		No		Total		
	*n*	%	*n*	%	*n*	%	
**Gender**							0.16 *
Male	65	67.7	226	60.0	291	61.3	
Female	31	32.3	151	40.0	182	38.5	
**Education**							0.46 *
Under high school	69	71.9	245	65.2	314	66.5	
High school	23	24.0	110	29.2	133	28.2	
Above high school	4	4.1	21	5.6	25	5.3	
**Marital status**							0.59 *
Single	18	18.8	56	14.9	74	15.6	
Live with spouse/partner	63	65.6	252	66.8	315	66.6	
Divorce/Widow	15	15.6	69	18.3	84	17.8	
**Employment**							<0.01 *
Unemployed	17	17.7	27	7.1	44	9.3	
Self-employed	46	47.9	268	70.9	314	66.2	
Employed	33	34.4	83	22.0	116	24.5	
**Study site**							<0.01 *
Thanh Hoa HIV/AIDS Control Center	35	36.6	103	27.2	138	29.1	
Quang Xuong District General Hospital	9	9.4	32	8.4	41	8.6	
Bao Thang District General Hospital	9	9.4	87	23.0	96	20.2	
Ung Hoa District Health Center	5	5.2	94	24.8	99	20.8	
Ba Vi District General Hospital	38	39.6	63	16.6	101	21.3	
	**Mean**	**SD**	**Mean**	**SD**	**Mean**	**SD**	***p*-Value**
**Age**	37.9	7.6	38.4	8.4	38.4	8.3	0.78 ^†^

* χ^2^ test; ^†^ Mann–Whitney test; significance level was *p* < 0.01.

**Table 2:** Antiretroviral therapy and substance abuse status (*n* = 482).

Characteristics	Depression						*p*-Value
	Yes		No		Total		
	*n*	%	*n*	%	*n*	%	
**HIV period**							0.04 *
Asymptomatic	69	76.7	308	84.2	377	82.7	
Symptomatic	19	21.1	43	11.7	62	13.6	
AIDS	2	2.2	15	4.1	17	3.7	
**Satisfied with treatment results**	80	85.1	345	94.8	425	92.8	<0.01 *
**Early ART initiation (cell/μL)**							0.65 *
<350	38	46.4	130	46.9	168	46.8	
350 ≤ CD4 cell count < 500	22	26.8	62	22.4	84	23.4	
≥500	22	26.8	85	30.7	107	29.8	
**Types of service**							0.10 *
Having HCT service	49	51.0	229	60.4	278	58.5	
No HCT service	47	49.0	150	39.6	197	41.5	
**Hazardous drinking**	28	29.2	121	31.9	149	31.0	0.60 *
**Binge drinking**	31	32.3	121	31.9	152	32.0	0.94 *
**Smoking**	59	61.5	195	51.6	254	53.6	0.08 *
**Drug user**	53	55.2	156	41.2	209	44.0	0.01 *
	**Mean**	**SD**	**Mean**	**SD**	**Mean**	**SD**	***p*-Value**
**ARV duration (year)**	3.3	2.5	3.9	2.5	3.8	2.5	0.02 ^†^
**Number of CD4 cells at the start of ART**	383	267	391	248	389	252	0.67 ^†^
**Number of CD4 cells at the last test**	444	265	461	237	455	244	0.47 ^†^

* χ^2^ test; ^†^ Mann–Whitney test; significance level was *p* < 0.01.

**Table 3:** Severity score of depression among patients receiving ART (*n* = 482).

Severity Score	*n*	%
**Major depressive syndrome**	96	20.2
**Depressive symptoms**		
None (0–4)	233	49.1
Mild (5–9)	146	30.8
Moderate (10–14)	61	12.8
Moderate severe (15–19)	22	4.6
Severe (≥20)	13	2.7

**Table 4:** Health-related quality of life among patients receiving ART (*n* = 482).

Characteristics	Depression						*p*-Value
	Yes		No		Total		
	*n*	%	*n*	%	*n*	%	
**Mobility**	60	63.2	78	20.0	138	29.4	0.01 *
**Self-care**	20	20.8	26	6.9	46	9.7	0.01 *
**Usual activities**	46	47.9	51	13.5	97	20.4	0.01 *
**Pain/discomfort**	71	74.0	157	41.4	228	48.0	0.01 *
**Anxiety/depression**	76	79.2	181	47.9	257	54.2	0.01 *
**Complication and concurrent disease**	53	55.2	120	31.7	173	36.4	0.01 *
	**Mean**	**SD**	**Mean**	**SD**	**Mean**	**SD**	***p*-Value**
**EQ5D index**	0.6	0.3	0.8	0.2	0.8	0.2	0.01 ^†^
**EQ-VAS**	62.1	14.8	76.4	14.1	73.6	15.3	0.01 ^†^

* χ^2^ test; ^†^ Mann–Whitney test; significance level was *p* < 0.01.

**Table 5:** Discrimination/stigma among patients receiving ART.

Characteristics	Depression						*p*-Value
	Yes		No		Total		
	*n*	%	*n*	%	*n*	%	
**Blamed because of health status**	43	45.3	124	32.8	167	35.3	0.02 *
**Shame because of health status**	54	56.3	157	41.5	211	44.5	0.01 *
**Discrimination in**							
Workplace	6	9.0	7	3.5	13	4.9	0.10 *
Health facilities	3	4.5	6	3.0	9	3.4	0.70 *
Family	5	7.5	8	4.0	13	4.9	0.32 *
Community	49	73.1	138	68.7	187	69.8	0.49 *
**Fear of HIV transmission during daily life and communication**	44	46.3	166	46.0	210	46.1	0.90 *
**Disclose health information with**							
Spouse/partner	51	54.8	234	64.5	285	62.5	0.08 *
Family	40	43.0	152	41.9	192	42.1	0.84 *
Relatives	16	17.2	62	17.1	78	17.1	0.90 *
Friends	11	11.8	44	12.1	55	12.1	0.90 *
Health workers	22	23.7	77	21.3	99	21.8	0.61 *
Peer educators	3	3.2	8	2.2	11	2.4	0.70 *
**Receive peers’ support**	45	56.3	175	61.6	220	60.4	0.39 *
**Share health information with peers**	50	62.5	179	60.3	229	60.7	0.71 *

* χ^2^ test; significance level was *p* < 0.01.

**Table 6:** Factors associated with depression among patients receiving ART.

Characteristic	Depression ^1^		PHQ-9 Score ^2^	
	OR	95%CI	Coef	95%CI
**Gender (Female vs. Male)**	0.37 *	0.12; 1.10		
**Marital status (Live with spouse/partner vs. Single)**	4.66 **	1.25; 17.42		
**HIV stage (Symptomatic vs. asymptomatic)**			4.50 ***	1.99; 7.00
**Number of CD4 cell at the start of ART (vs. ≥ 500)**				
350 ≤ CD4 cell count < 500	4.18 **	1.15; 15.21	0.44	−1.28; 2.12
<350	1.09	0.37; 3.25	0.08	−1.39; 1.55
**Type of services (no HCT vs. having HCT)**	8.37 ***	2.47; 28.34	1.39 *	−0.23; 3.00
**Satisfied with treatment results (Yes vs. No)**	0.18 *	0.03; 1.09	−4.35 ***	−6.82; −1.87
**AUDIT score**			0.29 **	0.07; 0.50
**Currently using drug (Yes vs. No)**	0.33	0.07; 1.45		
**Having the problems with mobility (Yes vs. No)**			2.62 ***	1.03; 4.21
**Having problems performing daily activities (Yes vs. No)**	1.65	0.61; 4.44	1.78 **	0.12; 3.45
**Pain/discomfort (Yes vs. No)**	7.23 ***	1.79; 29.15		
**Complication and concurrent disease (Yes vs. No)**			1.61 **	0.20; 3.02
**Suffer from discrimination in (Yes vs. No)**				
Work place	19.69 ***	2.55; 152.13	2.43	−0.48; 5.33
Health facilities	12.91 *	0.97; 171.12		
Community	3.57 **	1.20; 10.62	1.96 **	0.40; 3.51
**Disclose health status with (Yes vs. No)**				
Family			−1.46 **	−2.85; −0.06
Relatives	3.26	0.62; 17.18	2.88 **	0.68; 5.07
Health workers			−1.83 *	−3.98; 0.32
Peer educators	0.03 *	0.00; 1.94		

*** *p* < 0.01, ** *p* < 0.05, * *p* < 0.1; ^1^ Multivariate logistic regression model; ^2^ Multivariate Tobit regression model.

**Table 7:** Factors associated with health-related quality of life (HRQOL) among patients receiving ART.

Characteristics	Health-Related Quality of Life			
	EQ-5D-5L ^1^		EQ-VAS ^1^	
	Coef	95%CI	Coef	95%CI
**Age**	−0.01 ***	−0.01; −0.00	−0.14	−0.34; 0.07
**Education (High school and above vs. Under high school)**	−0.06 **	−0.13; −0.00		
**Employment (ref-Unemployed)**				
Self-employed	0.16 ***	0.06; 0.26	7.06 ***	1.99; 12.12
Employed	0.13 **	0.02; 0.24	5.28 *	−0.21; 10.77
**HIV stage (ref-asymptomatic)**				
Symptomatic	0.10 **	0.01; 0.19	5.74 ***	1.47; 10.01
AIDS	0.02	−0.15; 0.19	6.11	−2.50; 14.72
**Satisfied with treatment results (Yes vs. No)**			4.39	−0.86; 9.63
**Depression (Yes vs. No)**	−0.16 ***	−0.23; −0.10	−7.10 ***	−10.84; −3.36
**Drinking alcohol (AUDIT_C)**			0.36	−0.15; 0.87
**Drug users (Yes vs. No)**	−0.14 ***	−0.24; −0.05	−5.73 ***	−9.31; −2.16
**Smokers (Yes vs. No)**				
**Complications (ref-No)**	−0.10 ***	−0.16; −0.04	−7.34 ***	−10.56; −4.11
**Blamed because of health status (ref-No)**	−0.05 *	−0.11; 0.01		
**Suffer from discrimination (ref-No)**	−0.14 ***	−0.21; −0.07		
**Receive peers’ support (ref-No)**	−0.04	−0.12; 0.02		
**Constant**	1.12 ***	0.92; 1.31	75.30 ***	64.90; 85.70

*** *p* < 0.01, ** *p* < 0.05, * *p* < 0.1. ^1^ Multivariate Tobit regression model.
